# Six1 induces protein synthesis signaling expression in duck myoblasts mainly via up-regulation of *mTOR*

**DOI:** 10.1590/1678-4685-GMB-2015-0075

**Published:** 2016

**Authors:** Haohan Wang, Xinxin Li, Hehe Liu, Lingli Sun, Rongping Zhang, Liang Li, Mincheng Wangding, Jiwen Wang

**Affiliations:** 1 Institute of Animal Genetics and Breedings, Sichuan Agricultural University, Ya'an, China

**Keywords:** duck, Six1, protein metabolism signaling, mTOR, pEGFP-duSix1

## Abstract

As a critical transcription factor, *Six1* plays an important role in the regulation of myogenesis and muscle development. However, little is known about its regulatory mechanism associated with muscular protein synthesis. The objective of this study was to investigate the effects of overexpression of*Six1* on the expression of key protein metabolism-related genes in duck myoblasts. Through an experimental model where duck myoblasts were transfected with a pEGFP-duSix1 construct, we found that overexpression of duck*Six1* could enhance cell proliferation activity and increase mRNA expression levels of key genes involved in the PI3K/Akt/mTOR signaling pathway, while the expression of *FOXO1*, *MuRF1*and *MAFbx* was not significantly altered, indicating that*Six1* could promote protein synthesis in myoblasts through up-regulating the expression of several related genes. Additionally, in duck myoblasts treated with LY294002 and rapamycin, the specific inhibitors of*PI3K* and *mTOR*, respectively, the overexpression of *Six1* could significantly ameliorate inhibitive effects of these inhibitors on protein synthesis. Especially, the mRNA expression levels of *mTOR* and *S6K1* were observed to undergo a visible change, and a significant increase in protein expression of S6K1 was seen. These data suggested that *Six1*plays an important role in protein synthesis, which may be mainly due to activation of the mTOR signaling pathway.

## Introduction

*Six1* (Sine oculis homeobox homolog 1), which belongs to the sine oculs homeobox gene family, plays extensive roles in regulating the development of many tissues and organs, especially the development of skeletal muscle (Fougerousse *et al.*, 2002;Laclef *et al.*, 2003;Brodbeck and Englert, 2004; Brugmann *et al.*, 2004; Giordani *et al.*, 2007). Indeed, absence of the *Six1* gene caused mouse fetal death because of severe rib malformations and extensive muscle hypoplasia (Grifone *et al.*, 2005). Furthermore, Yajima *et al.* (2010) showed that overexpression of *Six1* in muscle satellite cells enhanced myoblast fusion and increased the number of nuclei per myotube. Intriguingly, myoblast fusion is a process similar to that occuring during muscle hypertrophy in adult skeletal muscle after overload (McCarthy*et al.*, 2011). In addition, Kostek *et al.* (2007) detected that*Six1* expression significantly decreased after 3 and 6 h post resistance training of human leg muscle. Until now, although a lot of research about the roles of *Six1* on skeletal muscle development and growth has been done, further studies on its regulatory mechanism involving muscle hypertrophy are still needed.

It is well known that skeletal muscle is high plastic in its organization, and its growth is known as a process of manifestation of externalization of muscle hypertrophy. This mainly depends on the ratio of muscular protein synthesis and degradation, and the size of muscular fiber increases when the rate of protein synthesis exceeds that of protein degradation. (Kimball, 2007; Tipton *et al.*, 2009). Recent studies have showed that the PI3K/Akt/mTOR signaling axis, considered as one of the crucial signaling cascades, isinvolved in skeletal muscle hypertrophy (Dorn 2nd and Force 2005; Dorn 2nd 2007;Bernardo *et al.*, 2010). Studies on swimming exercise-induced physiological hypertrophy in mouse heart revealed that *Six1* was significantly up-regulated, and that*Six1* and its cofactor *Eya2* could directly up-regulate the expression of *mTOR*, hence indicating that the Six1-Eya2 complex is a key regulator of physiological hypertrophy (Lee *et al.*, 2012). In addition, *Six1* can not only stimulate the activation of Akt and MAPK, but it can also up-regulate the expression of p-Akt (Yu *et al.*, 2006). Moreover, the promoter region of PI3K (p110α) contains putative *Six1* binding sites. These studies indicated that *Six1* plays a role in promoting muscle hypertrophy. However, whether *Six1* can regulate protein synthesis to facilitate muscle growth is still unclear.

It is generally believed that activation of PI3K is induced by insulin-like growth factor I (IGF1) (Musaro *et al*., 2001). When IGF1 binds to IGF1 receptor it can result in insulin receptor substrate-1 phosphorylation, followed by activation of PI3K and production of phosphatidylinositol-3,4,5-triphosphates, which subsequently recruit Akt to the plasma membrane and phosphorylate Akt, finally leading to the activation of mTOR and blockade of glycogen synthase kinase 3β (GSK3β). Furthermore, mTOR activity is mediated by direct phosphorylation of its downstream targets S6K1 and PHAS-1, which can ultimately result in protein synthesis. In contrast, Akt stimulation can domi-nantly inhibit the induction of atrophy signaling. As genetic activation of Akt can phosphorylate FOXOs, the translocation and activity of FOXO transcription factors can up-regulate the expression of Muscle Ring Finger1 (*MuRF1*) and Muscle Atrophy F-box (*MAFbx*), which encode E3 ubiquitin ligases, thus finally causing skeletal muscle protein degradation (Glass, 2005; Sandri, 2008). Protein synthesis and degradation in skeletal muscle being dependent on the changes in expression of PI3K/Akt/mTOR signaling pathways, it is of particular importance to study the regulatory mechanism between *Six1* and this signaling pathway.

In the present study aiming to investigate the effects of *Six1* on protein metabolism in duck myoblasts, we firstly determined the effects of overexpression of *Six1* on the expression levels of key genes related to protein metabolism signaling pathway. Furthermore, *in vitro* cultured duck myoblasts were treated *with*LY294002 and rapamycin, specific inhibitors of PI3K and mTOR, respectively, and were subsequently transfected with a pEGFP-duSix1 construct. Real-time RCR and western-blot methods were used to examine the expression levels of genes involved in the PI3K/Akt/mTOR signaling pathway. Our data showed that overexpression of*Six1* in myoblasts could significantly increase protein synthesis mainly via activation of the mTOR signaling pathway.

## Materials and Methods

### Animals

Hatching Peking duck (*Anas platyrhynchos domestica*) eggs were incubated at a temperature of 370.5°C and humidity of 86-87%. Eight eggs were obtained randomly after 13 days of incubation from the Sichuan Agricultural University Waterfowl Breeding Experimental Farm. All procedures were approved by the Guidelines on Humane Treatment of Laboratory Animals (2006).

### Cell culture

Primary myoblasts were isolated from the leg muscle of 13-day-old Peking duck embryos and purified using an improved technique of differential anchoring velocity and were seeded onto 6-well plates. The cells were cultured in Dulbecco's modified Eagle's medium-high glucose (DMEM) (Hyclone lab, Logan, UT), supplemented with 10% fetal bovine serum (FBS) (Gibco, New York) and kept at 37 °C in a humidified 5% CO_2_ incubator. A trypsin (0.25%)/EDTA solution was used to detach primary myoblasts from the culture plates for subculture (Continous cell culture).

### Cell treatments and transfection

Duck myoblast cells were seeded into 6-well plates at a density of 110^6^ cell/plate and incubated in DMEM supplemented with 10% FBS for 24 h at 37 °C in 5% CO_2_ incubator. Cells were then treated with or without LY294002 (Beyotime, Shanghai, China) (30μM and 40μM), and after treating the cells for 12 h and 24 h, respectively, these were washed twice with 1 mL of Hanks' balanced salt solution and harvested for subsequent real-time PCR and western-blotting analysis. The same method was used to treat cells with or without rapamycin (Beyotime, Shanghai, China) at 30nM and 40nM doses. Moreover, the cells were treated with LY294002 or rapamycin for proper time, followed by two washes with 1 mL of Hanks' balanced salt solution and then transfected with the duck recombinant pEGFP-duSix1 vector (Wang*et al.*, 2014) with or without 10 mmol/l 4-PBA for 24 h and 48 h using lipofectin 2000 (Invitrogen, USA) following the manufacturer's instructions. At the end of the incubation period, cells were harvested, and cell lysates were immediately frozen at −80°C for subsequent analysis.

### MTT assay

The MTT (3-(4,5-dimethylthiazol-2-yl)-2,5-diphenyltetrazolium bromide) assay, which measures cell proliferation and cytotoxicity, was used to analyze myoblast proliferative activity. Subcultured myoblasts (~1000/well) were seeded into 96-wells plates and incubated for 12, 24 or 48 h at 37°C. MTT (10ul, 5mg/ml, Sigma, China) was added into the culture system for 4 h at 37°C. The solutions were then replaced with 150ul of dimethyl sulfoxide (DMSO, Sigma, China). After a 20 min incubation, the absorbance value of each well was measured using a microplate reader at a wave length of 570 nm.

### Real-time PCR analysis

Total RNA was extracted from cells using Trizol (Invitrogen, USA) following the manufacturer's instructions. RNA concentration and purity were determined by spectrophotometric absorbance at 260 nm and 280 nm, and RNA integrity was detected by visualization of the 28S and 18S ribosomal subunits after electrophoresis in a 1.5% agarose gel. The SYBR Prime Script RT-PCR Kit (TaKaRa, Japan) was used for real-time PCR detection. Real-time PCR primers (Table 1) were designed for duck*Six1* (GenBank Accession No: KC990828.1), *PI3K, Akt, mTOR, S6K1, FOXO1, MAFbx, MuRF1* and for the detection of two reference genes, β-actin (GenBank Accession No: EF667345.1) and*GAPDH* (GenBank Accession No: GU564233.1). PCR assays were carried out with CFX96 real-Time PCR Detection System (Bio-Rad, CA, USA) in 96-well plates. Reactions mixtures of 25 μl contained 1 μl of cDNA template, 12.5 μl of SYBR Premix Ex Taq, 10.5 μl of sterile water, and 0.5 μl of each gene specific primer (Table 1). The procedure for real-time PCR was as follows: 30 s of pre-denaturationreaction at 95°C, followed by 40 cycles of 95°C for 10 s, 60°C for 40 s and 72°C for 20 s, and finally a melting curve analysis program with 60-95°C with a heating rate of 0.1°C per second and continuous fluorescence measurement. All PCRs were performed in triplicate for each sample, with tubes without cDNA as negative controls.

**Table 1:** Primers used for quantitative real-time PCR used in this study.

Gene name	Primer name	Primer sequence (5-3)	Product size (bp)	Annealing temperature(°C)
*Six1*	Q-Six1F	GATGCTGCCGTCGTTTGG	144	60
	Q-Six1R	AGGACGCTCTCGTTCTTGTG		
*PI3K*	Q-PI3KF	CTTTTACCGAGGAGGTTCTGTGG	137	60
	Q-PI3KR	CTGAAGGTTGGTCTTTGTGGAC		
*Akt*	Q- Akt F	TCTTTGCTGGCATTGTTTGGC	152	60
	Q- Akt R	GCTGTCATCTTGGTCAGGAGGAGT		
*mTOR*	Q- mTOR F	CTATCTGCCTCAGCTCATTCCT	121	60
	Q- mTOR R	GTCATCCAGGTTAGCTCCAAAG		
*S6K1*	Q- S6K1F	ATAATCGTGCTGTGGACTGGTG	155	60
	Q- S6K1R	TCTGGCTTCTTGTGTGAGGTAGG		
*FOXO1*	Q- FOXO1F	AGGTTCACCAAATCCAGACTACAGG	182	60
	Q- FOXO1R	CGTTGTGCGGAGGAGAATCAG		
*MAFbx*	Q- MAFbxF	CAGACCCTCTACACATCCCTCT	123	60
	Q- MAFbxR	GTTCAGTTGCTGTTGCCAGTG		
*MuRF1*	Q- MuRF1F	TCAACATCTACTGCGTCACCTG	128	60
	Q- MuRF1R	GCTATTCAACTCGCTCTTCTGG		
β*-actin*	Q-β-actinF	GCTATGTCGCCCTGGATTTC	168	60
	Q-β-actinR	CACAGGACTCCATACCCAAGAA		
*GAPDH*	Q-GAPDHF	AAGGCTGAGAATGGGAAAC	254	60
	Q-GAPDHR	TTCAGGGACTTGTCATACTTC		

### Western-blotting

Whole protein from cells of different treatments was exacted by disruption in RIPA buffer (Sigma-Aldrich, St. Louis, MO, USA) in the presence of protease inhibitor cocktail. The lysates were kept on ice for 1 h, and then centrifuged at 12000 g for 20 min at 4°C. The resulting supernatants were collected for western blot analysis. Protein concentrations were determined by the BCA protein assay (Beyotime, Shanghai, China) following the manufacturer's instructions. Approximately 15 |ig protein of each supernatant fraction was mixed with 6SDS sample buffer and boiled for 5 min. Subsequently, the proteins were separated by 10% SDS-polyacrylamide gel electrophoresis (SDS-PAGE), and then transferred to a PVDF membrane (Milli-Pore, Bedford, MA, USA). The membranes were blocked in 5% non-fat dry milk in TBST (5 mmol Tris-HCl, pH 7.4, 136 mmol NaCl, 0.05% Tween20) for 3 h at room temperature and then incubated with primary antibodies against FOXO1 (L27, Source:Rabbit), p-FOXO1 (Ser256, Source:Rabbit), S6K1(Source:Rabbit), and Tubulin at a 1:1000 dilution overnight at 4°C. These antibodies were purchased from Cell Signaling Technology (Beverly, MA, USA). The next day, the membranes were washed three times for 15 min with TBST, followed by incubation in horse-radish peroxidase (HRP)-conjugated goat anti-IgG rabbit (dilution 1:500, Beyotime, Shanghai, China) for 3 h at room temperature and washed three times with TBST. Each sample was analyzed in triplicate. Antibody binding was detected by electro-chemiluminescence using fluorescence detection equipment (ChemiDoc XRS; Bio-Rad, Hercules, CA, USA).

### Statistical analysis

The relative gene expression levels were analyzed and estimated using the comparative Ct (2^−ΔΔCt^) value method. Western-blot pixel densities between different treatment groups were analyzed by Student's *t*-test. All statistical analyses were performed using Microsoft Excel software and SAS version 9.13. Comparisons of the group means were run by one-way ANOVA and Duncan's multiple range test was used to analyze statistical significance. All results were shown as means ± SEM. A P < 0.05 was considered statistically significant.

## Results

### The influence of overexpression of duck Six1 on expression of key genes involved in the PI3K/Akt/mTOR signaling pathway

**Figure 1:**
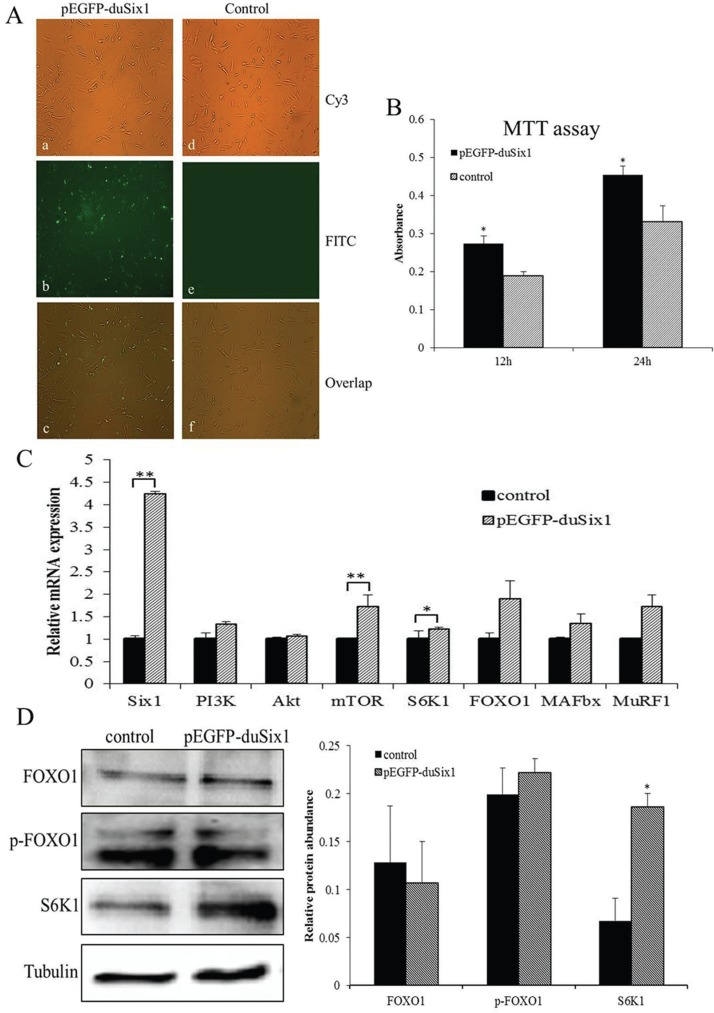
Effects of overexpression of *Six1* on protein synthesis and degradation in duck myoblasts. A: The expression of GFP in duck myoblasts after transfection of *Six1* (100). Notes: (a), (b) and (c) are pEGFP-duSix1-transfected myoblasts and (d), (e) and (f) are non-transfected myoblasts (control). (a) and (d) showed the shape of the myoblasts in the Cy3 channel; (b) and (e) showed the green fluorescent protein in the FITC channel; (c) and (f) show the overlap of (a) with (b) and (d) with (e), respectively. B: Effects of Six1-transfection on the cellular proliferation of duck myoblasts at 12 h and 24 h after transfection. Cellular proliferation was measured by colorimetric MTT assay. Absorbance was measured at 570 nm, and data are presented as means ± SEM, n = 5 wells. C: Relative expression levels of*Six 1* and the key genes related to protein synthesis and degradation in duck myoblasts after transfection. Duck P-actin and *GAPDHwere* used as the internal controls. **(P < 0.01) and *(P < 0.05), values are means ± SEM, n = 3 for each group. D: Western blot analysis showing the effect of Six1-transfected on protein expression of FOXO1, p-FOXO1, S6K1 in duck myoblasts. Bar graphs showing quantification of the expression level of each protein compared with the expression of tubulin. Data are expressed as means ± SEM. Western blots are representative of three independent experiments. Western immunoblots probed with antibodies against FOXO1, phospho-FOXO1, S6K1 and tubulin were used as internal controls.

Duck myoblasts were transfected with the eukaryotic expression vector pEGFP-duSix1. Figure 1A shows that green fluorescence protein (GFP) was markedly expressed in Six1-transfected cells, indicating that *Six1* was also expressed in duck myoblasts. Furthermore, results from the MTT assay showed that overexpression of*Six1* could stimulate cellular proliferation at 12 h and 24 h after transfection, exhibiting a significant increase in the absorption value at 570 nm, and a higher cell proliferative activity at 24 h (Figure 1B).

Cells transfected with pEGFP-duSix1 for 24 h and were harvested to detect both mRNA and protein levels of key genes related to protein synthesis and degradation. Results of real-time PCR showed that overexpression of duck*Six1* could influence both protein synthesis and degradation in myoblasts. Especially, mRNA expression levels of *mTOR* and its downstream effector, *S6K1*, which are central regulators of protein synthesis, were significantly increased (P < 0.05) (Figure 1C). The expression levels of*PI3K* and *Akt* mRNA displayed an increasing but not significant trend (P > 0.05). Similar to *PI3K* and*Akt*, the mRNA expression levels of *FOXO1, MuRF1* and *MAFbx*, which are related to protein degradation, were also not significantly changed (P > 0.05). These results indicate that *Six1* may play an important role in protein synthesis.

Similar to mRNA levels and protein phosphorylation status we observed no insignificant change in protein expression of FOXO1 after transfection. However, the protein expression of S6K1, which is a downstream effector of PI3K and mTOR required for positively regulating protein synthesis, was greatly increased by overexpression of *Six1* (P < 0.05) (Figure 1D). All the results of real-time PCR and western-blot immunoassay are in supported that *Six1* plays an important role in regulating protein synthesis.

### The effect of overexpression of duck Six1 on protein synthesis of myoblasts was blocked by LY294002

**Figure 2:**
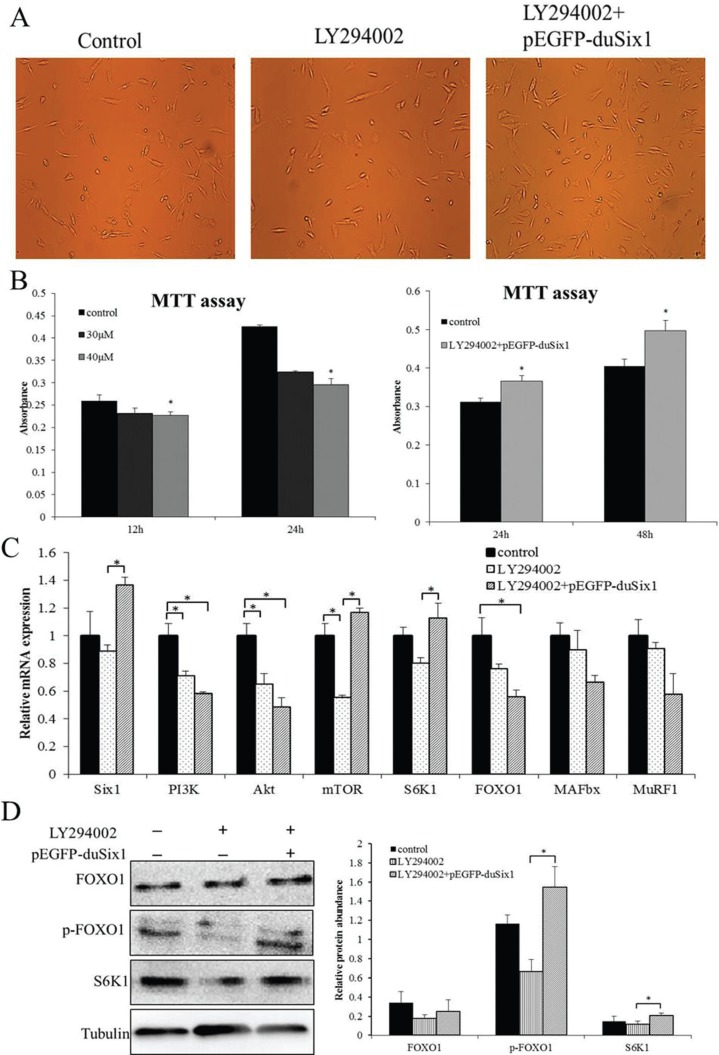
Overexpression of *Six1* can stimulate protein synthesis in LY294002-treated duck myoblasts. A: Representative images of myoblasts for different treatments groups, (a) control cells, (b) LY294002 treatment cells at 24 h, (b) LY294002 treatment cells for 24 h and then transfected with pEGFP-duSix1for 48 h. B: Effects of different treatments on cell proliferation of duck myoblasts at different times. Cell proliferation was measured by MTT assay. Absorbance was measured at 570 nm, and data are presented as means ± SEM, n = 5 wells, *(P < 0.01). C: Relative expression levels of *Six1* and key genes related to protein synthesis and degradation in duck myoblasts of different treatments. Duck P-actin and *GAPDHwere* used as internal controls. **(P < 0.01) and *(P < 0.05), values are means ± SD, n = 3 for each group. D: Western blot analysis showing the effects of transfection of *Six1* on LY294002 treatments in duck myoblasts. Bar graphs showing quantification of the expression level of each protein compared with the expression of tubulin. Data are expressed as means ± SEM. Western blots are representative of three independent experiments. Western immunoblots probed with antibodies against FOXO1, phospho-FOXO1, S6K1 and tubulin were used as internal controls.

According to previous research, Six1 may bind to the promoter region of PI3K (p110α), indicating that a potential regulatory relationship may exist between Six1 and PI3K signaling. To further elucidate whether Six1 regulates the activation of PI3K signaling to affect protein synthesis, stable growth duck myoblasts were incubated in the presence or absence of LY294002, which is a highly potent and specific inhibitor of PI3K. In order to seek a proper time and concentration of LY294002 treatment, an MTT assay was used to test cell proliferative activity at 12 h and 24 h after treatment with 30 μM and 40 μM LY294002, respectively. As shown in Figure 2B, cell proliferative activity was significantly inhibited by LY294002. Moreover, the higher concentration showed more obvious inhibition of cell proliferation and more serious inhibitive effects at 24 h than at 12 h (Figure 2B). Subsequently, we transfected the LY294002- treated cells with pEGFP-duSix1 for 24 h and 48 h, respectively, and found that overexpression of duck *Six1* could significantly increase cell proliferation activity after transfection (Figure 2B).

To verify our model of LY294002-treatment and to contextualize our expression results, relative expression levels of key genes were measured as previously experiment. Real-time PCR showed that *PI3K* expression was significantly reduced in the presence of LY294002 (Figure 2C), suggesting that LY294002 could significantly inhibit expression of PI3K. The lower PI3K activity was also associated with a decrease in expression levels of its downstream genes, including *Akt, mTOR, S6K1* which relate to protein synthesis, and *FOXO1, MuRF1, MAFbx* which are associated with protein degradation. Moreover, to determine whether overexpression of *Six1* would improve the expression levels of these genes, the LY294002-treated cells were transfected with pEGFP-duSix1 for 48 h, as shown in Figure 2C. The expression of *Six1* was notably increased (P < 0.05), whereas *PI3K* and *Akt* were not changed (P > 0.05). A similar trend in mRNA expression levels was also observed for *FOXO1* and its downstream targets *MuRF1, MAFbx*. Contrary to the expression levels of *PI3K*and *Akt*, an increase in mRNA levels was seen for mTOR and*S6K1* in myoblasts, indicating that overexpression of*Six1* may up-regulate *mTOR* expression to promote protein synthesis and ultimately to rescue the inhibitive effects of LY294002.

The expression levels of key proteins mentioned above were also examined by western-blot immunoassay, and the results revealed that the expression of key proteins was in accordance with mRNA levels, displaying a trend to reduced expression levels of FOXO1, p-FOXO1 and S6K1 protein after treatment with LY294002 in myoblasts. In contrast, after transfection with Six1 for 48 h, total FOXO1 protein levels were not changed, while its phosphorylation and S6K1 protein levels showed a great increase, suggesting that overexpression of*Six1* could promote activation of S6K1 and induce protein synthesis to rescue the inhibitive effects of LY294002.

### The effects of overexpression of duck Six1 on protein synthesis of myoblasts were blocked by rapamycin

**Figure 3:**
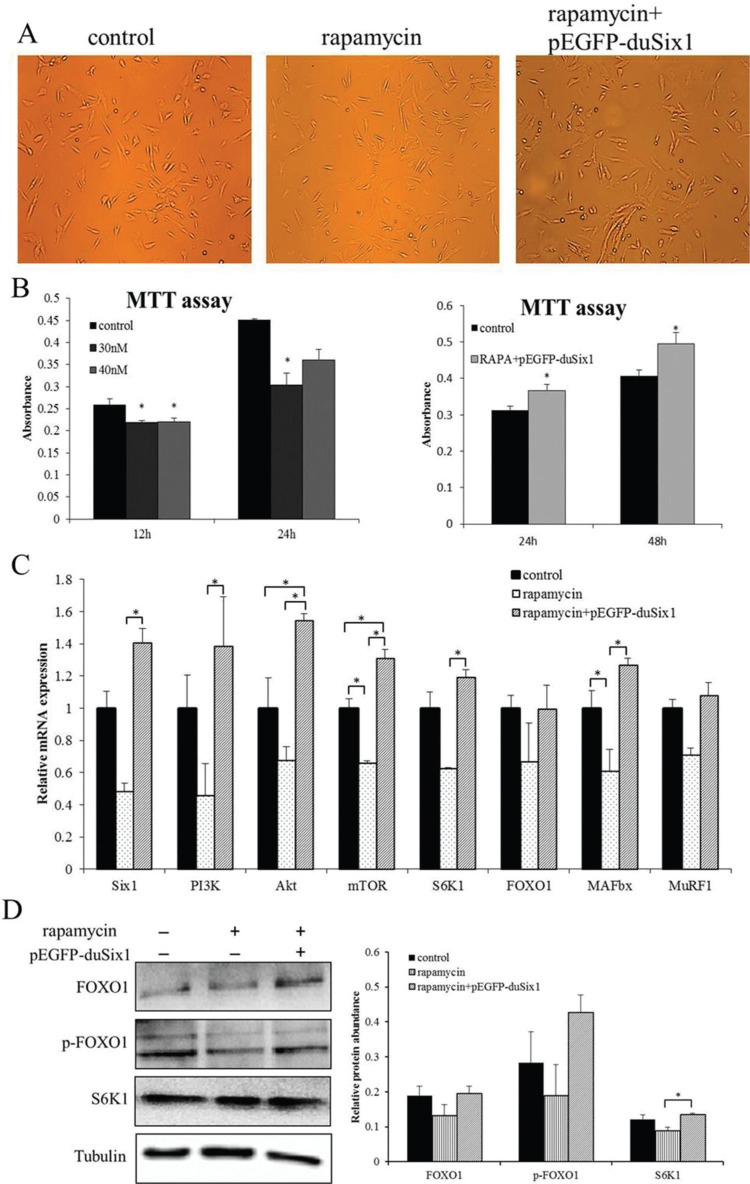
Overexpression of *Six1* can stimulate protein synthesis in rapamycin-treated duck myoblasts. A: Representative images of myoblasts for different treatments groups, (a) control cells, (b) rapamycin treatment cells at 24 h, (c) Rapamycin treatment of cells for 24 h was followed by transfection with pEGFP-duSix1 for 48 h. B: Effects of different treatments on cell proliferation of duck myoblasts at different times. Cell proliferation was measured by MTT assay. Absorbance was measured at 570 nm, and data are presented as means ± SEM, n = 5 wells, *(P < 0.01). C: Relative expression levels of*Six1* and key genes related to protein synthesis and degradation in duck myoblasts after different treatments. Duck β-actin and *GAPDH* were used as the internal controls. **(P < 0.01) and *(P < 0.05), values are means ± SEM, n = 3 for each group. D: Western blot analysis showing the effects of transfection of*Six1* on rapamycin treatments in duck myoblasts. Bar graphs showing quantification of the expression level of each protein compared with the expression of tubulin. Data are expressed as means ± SEM. Western blots are representative of three independent experiments. Western immunoblots probed with antibodies against FOXO1, phospho-FOXO1, S6K1 and tubulin were used as internal controls.

From our previous experiments we saw that over-expression of*Six1* could increase expression levels of protein synthesis-related genes. Interestingly, in LY294002-treated cells which were transfected with pEGFP-duSix1, the mRNA expression of *PI3K* was not significantly changed, while *mTOR* and its downstream*S6K1* were increased significantly. Previous studies had revealed that *Six1* and its cofactor Eya2 could simulate heart hypertrophy through directly up-regulating *mTOR* (Lee *et al.*, 2012). Therefore, to clarify whether overexpression of *Six1* simulates myoblasts protein synthesis via *mTOR*, cells were treated with rapamycin (the mTOR inhibitor) at different times and concentrations, as described above. As shown in Figure 3B, the results of the MTT assay indicated that cell proliferation activity was significantly decreased in the presence of this inhibitor, and that inhibition was more serious with prolonged treatment time, but not with the higher concentration of rapamycin. However, after transfecting the rapamycin-treated cells with pEGFP-duSix1 the cell proliferative activity was significantly increased (P < 0.05).

To validate whether overexpression of *Six1* greatly increases*mTOR* expression in the presence of 30 nM rapa-mycin and ultimately restores protein synthesis of myo-blasts, the same detection methods were performed in this study. Similar to the previous results, real-time PCR showed that relative expression of *mTOR* was significantly decreased after rapamycin treatment and the same trends were also found in other genes (Figure 3C), indicating that rapamycin could significantly inhibit the activation of mTOR, and finally led to weaken the protein synthesis in myoblasts. In addition, as shown in Figure 3C, over-expression of*Six1* could significantly up-regulate the expression levels of *mTOR* and its downstream effector *S6K1*. However, inconsistent with the previous experiment was the finding that relative expression levels of *PI3K*, *Akt* were increased significantly compared with rapamycin treatment. Moreover, expression levels of*FOXO1*, *MuRF1*, which is involved in protein degradation, showed insignificant changes after transfection with pEGFP-duSix1. These results provide the evidence that overexpression of *Six1*can significantly improve protein synthesis, mainly due to activation of*mTOR* signaling.

At the protein level, after treating myoblast cells with rapamycin, insignificant changes were found in protein expression of FOXO1, p-FOXO1 and S6K1 (P > 0.05) (Figure 3D), indicating that protein synthesis was inhibited by rapamycin. In addition, the effects of overexpression of *Six1* on the rapamycin-treated myoblasts were also investigated, and a significant increase in protein expression of S6K1 (P < 0.05) was seen. Moreover, as activation of S6K1 is directly mediated by mTOR, these results indicate that overexpression of *Six1* might promote protein synthesis by inducing the activation of mTOR to directly affect the translation machinery.

## Discussion

### Six1 stimulates protein synthesis via PI3K/Akt/mTOR signaling pathway

Six1 was initially considered as a critical transcription factor involved in the formation of eyes and other tissues. Furthermore, numerous studies have pointed out that *Six1* is an important regulator required for muscle development (Fougerousse *et al.*, 2002; Laclef *et al.*, 2003: Grifone*et al.*, 2005), muscle fiber differentiation (Grifone *et al.*, 2004), and tumorigenesis (Yu*et al.*, 2006; Farabaugh *et al.*, 2012). Additionally, another study indicated that *Six1* is also related to heart muscle hypertrophy (Lee *et al.*, 2012). To further understand the role of *Six1* on PI3K/Akt/mTOR signaling pathway in myoblasts, *Six1* was overexpressed by transfecting duck myoblasts with the eukaryotic expression vector pEGFP-duSix1. Similar to previous research, over-expression of*Six1* could significantly increase protein synthesis by up-regulating the mRNA expression of key genes involved in protein synthesis. Especially, overexpression of *Six1* resulted in an obvious increase in the mRNA expression levels of *mTOR* and both mRNA and protein levels of its downstream *S6K1*. However, the expression levels of muscle atrophy-related signaling genes were not significantly inhibited after transfection for 24 h.

Previous studies have shown that the mRNA expression levels of*MAFbx* and *MuRF1* were increased in atrophic skeletal muscle (Lecker *et al.*, 2004), but were inhibited by the activation of Akt/mTOR signaling*in vivo* (Bodine *et al.*, 2001). However, Leger *et al.* (2006) have reported that the mRNA and protein levels of MAFbx and MuRF1 were increased in hypertrophied muscle after 8 weeks of hypertrophy-inducing resistance training. Similarly, running for 30 min at a moderate-high intensity of 75% of V_O2max_ resulted in an increase in both *MAFbx* and *MuRF1* mRNA expression after 1-4 h of exercise (Louis *et al.*, 2007). Therefore, these studies suggested that the regulation of *MAFbx* and *MuRF1* may depend on the manner and intensity of exercise. Our results showed that over-expression of*Six1* did not significantly change *FOXO1*,*MAFbx* and *MuRF1* mRNA expression, and one possible explanation might be the short time of transfection and the transfection efficiency. An alternative explanation could be that overexpression of *Six1* can affect the cell cycle (Ford *et al.*, 2000), the regulation of which is also implicated in the activation of E3 ubiquitin-protein ligase.

### Overexpression of Six1 in myoblasts promotes protein synthesis mainly via activation of mTOR

As stated above, overexpression of *Six1* could significantly promote cell protein synthesis through up-regulating expression of key genes related to protein synthesis. Moreover, PI3K, as an upstream regulator of this signaling pathway, plays a critical role in protein synthesis and can promote muscle hypertrophy. To further uncover whether *PI3K* is involved in *Six1*-mediated regulation of muscular protein metabolism, LY294002, a specific inhibitor of the PI3K/Akt signaling targeting the catalytic site of p110 of PI3K (Liu *et al.*, 2008), was used in this study. It is well known that LY294002 can inhibit the activation of PI3K and ultimately regulate cell proliferation and cell cycle in a variety of cells, such as neutrophils, endothelial cells, and breast cancer cells (Dufourny *et al.*, 1997; Kanda *et al.*, 1997; Pellegatta *et al.*, 1998). Likewise, our results showed that LY294002 cannot only significantly decrease cell proliferation, but also significantly inhibit cellular protein biosynthesis by down-regulating the expression of genes involving the PI3K/AKT signaling pathway. Nevertheless, the results showed that the ubiquitin-proteasome pathway was not obviously up-regulated, showing only an insignificant fluctuation in mRNA levels and FOXO1 protein phos-phorylation. Akt can phosphorylate FOXOs and subsequently promote the shift of FOXOs from the nucleus to the cytoplasm. However, phosphorylation of Akt can be blocked by LY294002, then resulting in diminished levels of phosphorylated FOXOs in the cytoplasm and a marked increase in nuclear FOXO proteins, which finally leads to protein degradation via activation of ubiquitin-related signaling. Besides the signaling pathway of protein metabolism, there is still evidence that nutritional support also plays an important role in regulating protein synthesis, as protein degradation may be reduced following gradual consumption of the nutrient substances of cell-culture medium in order to maintain the balance of the cellular protein metabolism (Sakurai *et al.*, 1995). Moreover, the short duration of LY294002 treatment may also be a cause for the unchanged expression of protein degradation signaling pathway.

The effects of rapamycin on cellular protein metabolism were also investigated in duck myoblasts. Rapamycin is a selectively inhibitor of mTOR, as it can bind to members of the FK binding protein (FKBP) family, and the complex rapamycin/FKBP can then bind to mTOR and finally block its activity (Fang *et al.*, 2001; Foster, 2007). A similar result was found as previously described, where rapamycin could significantly decrease cell proliferation and activate mTOR/S6K1 signaling, which plays a critical role in protein synthesis. Previous studies revealed that rapamycin functions by mainly inhibiting the mTORC1 (one complex of mTOR), but long-term rapamycin treatment *in vitro* can also inhibit the mTORC2 complex and potentially affect Akt signaling by inducing the expression of *Akt* (Sarbassov *et al.*, 2006). Furthermore, the mTORC1 complex negatively regulates the IGFI pathway via S6K1 (Um *et al.*, 2004;Aguilar *et al.*, 2007). Interestingly, we found that expression of *PI3K*also decreased after treatment of rapamycin. Similarly, intermittent swimming training and injection with rapamycin in mice for two weeks led to a significant inhibition of PI3K, which may be mediated by a feedback regulation though mTOR/S6K1. Accordingly, the decreased mRNA levels of key genes related to cellular protein degradation might be due to reduced nutrient availability and/or the duration of rapamycin treatment.

In the present study, our data indicate that transfection with pEGFP-duSix1 would arrest the activity of PI3K/Akt/mTOR signaling pathway, thus attenuating the inhibitory effects of LY294002 and/or rapamycin in duck myoblasts. Interestingly, it was predicted that the promoter region of PI3K (p110α) contains putative *Six1* binding sites, suggesting that Six1 may play a regulatory role on *PI3K.* However, we found that transfecting myoblasts with pEGFP-duSix1 in combination with LY294002 treatment did not change the expression of *PI3K* and *Akt*. Furthermore, the PI3K/Akt pathway is mainly negatively regulated by many other anti-oncogenes, such as PTEN (Choi *et al.*, 2002), CTMP (Maira*et al*, 2001) and PHLPP (Gao *et al*, 2005), to inhibit tumor cell proliferation. These findings indicate that *Six1* may not directly regulate the expression of *PI3K*, and insignificant changes in *PI3K* and *Akt* expression may result from the effect of other regulator(s). It has been demonstrated that*Six1* and its cofactor *Eya2* could directly up-regulate *mTOR* (Lee*et al.*, 2012), which is consistent with our results where the expression of *mTOR* and *S6K1*were markedly enhanced after transfection. However, transfecting myoblasts with pEGFP-duSix1 in the presence of rapamycin led to increased expression levels of key genes associated with protein synthesis, which supports the hypothesis that*Six1* can regulate protein synthesis via activation of mTOR. Additionally, the expression of key genes associated with protein breakdown showed an increasing tendency. FOXO1 is regarded as an atrophy gene and acts as a sensor to induce muscle atrophy (McLoughlin*et al*, 2009). The increasing expression level of FOXO1 protein indicated that, after transfection with pEGFP-duSix1, cellular protein degradation was also increased. The different mRNA expression levels of*FOXO1, MAFbx* and *MuRF1* may, however, be due to the different inhibitor treatment, and more in-depth analyses are needed to address this issue.

In conclusion, the results of this present study showed that overexpression of duck *Six1* can increase protein synthesis, possibly by stimulating muscle hypertrophy-related signaling molecules, while the mRNA levels of key genes related to protein breakdown showed a small but not significant increase after transfection. Furthermore, we treated duck myoblasts with LY294002 and rapamycin (the specific inhibitor of PI3K and mTOR, respectively), and found that these inhibitors can significantly inhibit cellular protein synthesis. Overexpression of duck *Six1* could thus ameliorate the inhibitive effects, and *Six1* may up-regulate the expression of *mTOR* but not*PI3K* to promote protein synthesis. Nonethless, more in-depth research is needed to test this supposition. Taken together, it can be concluded that *Six1* can be regarded as a critical candidate gene to immediately regulate protein synthesis and is important for muscle hypertrophy in avian species.
